# Clinical implementation of plasma cell-free circulating tumor DNA quantification by digital droplet PCR for the monitoring of Ewing sarcoma in children and adolescents

**DOI:** 10.3389/fped.2022.926405

**Published:** 2022-08-15

**Authors:** Markus G. Seidel, Karl Kashofer, Tina Moser, Andrea Thueringer, Bernadette Liegl-Atzwanger, Andreas Leithner, Joanna Szkandera, Martin Benesch, Amin El-Heliebi, Ellen Heitzer

**Affiliations:** ^1^Division for Pediatric Hematology-Oncology, Department of Pediatrics and Adolescent Medicine, Medical University of Graz, Graz, Austria; ^2^Diagnostic and Research Center for Molecular BioMedicine, Diagnostic and Research Institute of Pathology, Medical University of Graz, Graz, Austria; ^3^BioTechMed-Graz, Graz, Austria; ^4^Diagnostic and Research Center for Molecular BioMedicine, Diagnostic and Research Institute of Human Genetics, Medical University of Graz, Graz, Austria; ^5^Christian Doppler Laboratory for Liquid Biopsies for Early Detection of Cancer, Medical University of Graz, Graz, Austria; ^6^Department of Orthopedics and Trauma, Medical University of Graz, Graz, Austria; ^7^Division of Oncology, Department of Internal Medicine, Medical University of Graz, Graz, Austria; ^8^Division of Cell Biology, Histology and Embryology, Gottfried Schatz Research Center, Medical University of Graz, Graz, Austria; ^9^Center for Biomarker Research in Medicine (CBmed), Graz, Austria

**Keywords:** cell-free DNA (cfDNA), minimal residual disease (MRD), circulating cell-free tumor DNA (ctDNA), Ewing sarcoma (EWS), pediatric oncology, ddPCR assay, breaktracer

## Abstract

**Background:**

Treatment stratification and response assessment in pediatric sarcomas has relied on imaging studies and surgical/histopathological evidence of vital tumor cells. Such studies and evidence collection processes often involve radiation and/or general anesthesia in children. Cell-free circulating tumor DNA (ctDNA) detection in blood plasma is one available method of so-called liquid biopsies that has been shown to correlate qualitatively and quantitatively with the existence of vital tumor cells in the body. Our clinical observational study focused on the utility and feasibility of ctDNA detection in pediatric Ewing sarcoma (EWS) as a marker of minimal residual disease (MRD).

**Patients and methods:**

We performed whole genome sequencing (WGS) to identify the exact breakpoints in tumors known to carry the *EWS-FLI1* fusion gene. Patient-specific fusion breakpoints were tracked in peripheral blood plasma using digital droplet PCR (ddPCR) before, during, and after therapy in six children and young adults with EWS. Presence and levels of fusion breakpoints were correlated with clinical disease courses.

**Results:**

We show that the detection of ctDNA in the peripheral blood of EWS patients (i) is feasible in the clinical routine and (ii) allows for the longitudinal real-time monitoring of MRD activity in children and young adults. Although changing ctDNA levels correlated well with clinical outcome within patients, between patients, a high variability was observed (inter-individually).

**Conclusion:**

ctDNA detection by ddPCR is a highly sensitive, specific, feasible, and highly accurate method that can be applied in EWS for follow-up assessments as an additional surrogate parameter for clinical MRD monitoring and, potentially, also for treatment stratification in the near future.

## Introduction

Ewing sarcoma (EWS) is a small round cell sarcoma with a yearly incidence rate of 2.4–3 per million children and young adults with a median age of 15 years at diagnosis. Unlike carcinomas and other malignancies, the mutational burden of pediatric sarcomas is low, and few recurring, disease-specific hotspot mutations in other driver genes have been reported (1–3). Instead, the key driver in EWS are oncogenic gene fusions involving one member of the FET family of genes (usually EWSR1, located on chromosome 22q12) and a member of the ETS family of transcription factors (4–7). The breakpoint of *EWSR1-ETS* is not conserved and varies in every individual tumor/patient. Yet, such fusions are reliably present throughout the clonal evolution of the tumor irrespective of additional somatic events that may occur.

EWS tends to disseminate early and already exhibit (micro-) metastases at diagnosis. It is a radiosensitive tumor that is treated by a combination of multi-agent chemotherapy, surgery, and/or radiotherapy, with a 5-year overall survival of less than 70% (8), with better outcomes in localized and worse (<10–30%) in primarily disseminated disease (9). Currently, due to a lack of plasma tumor markers, monitoring during the clinical course of the disease and post-therapeutic screening schedules depends on imaging studies, which involve anesthesia in younger children. To date the histopathological evaluation of suspect lesions using an image-guided or open biopsy is state of the art to confirm a recurrent or metastatic EWS.

In the last decade, liquid biopsies evolved as a potential companion to invasive biopsy and imaging and were shown to facilitate the diagnosis, monitoring, and tracking of minimal residual disease (MRD) (10, 11). Among the broad range of tumor-related biomarkers in a liquid biopsy, cell-free circulating tumor DNA (ctDNA) is thought to be the most promising candidate for a wide-spread clinical use (12). ctDNA-based approaches were demonstrated to yield a representative genetic picture of the primary tumor and potential secondary sites. Another major advantage of liquid biopsies over tissue biopsies is that liquid biopsies methods can be repeatedly applied to study the temporal tumor evolution from its primary site to metastases during the treatment course and follow-up. Therefore, ctDNA-based approaches represent a powerful tool for molecular profiling and tumor monitoring.

Recently, researchers expanded ctDNA approaches to other tumor-specific signals than genetic alterations and focused on methylation, fragment length distribution or nucleosome occupancy patterns to obtain a more comprehensive signature of the tumor (13–15). This may enhance biological research on step-wise dedifferentiation of tumors and the identification of novel treatment targets. Although initial studies demonstrating clinical applicability focused on advanced tumor stages, in which ctDNA levels are often higher compared to early stages, recent advances in molecular genetic technologies now allow the detection of minute amounts ctDNA in the blood plasma with high validity (reviewed in 10, 11). These technological improvements make ctDNA a promising marker for MRD testing and disease follow up. In particular, tumor-informed ctDNA approaches, i.e., the analysis of patient-specific, genetic alterations in blood plasma, can reach a high sensitivity. A number of studies highlighted the clinical utility of ctDNA monitoring during clinical care to facilitate minimal/measurable disease monitoring in pediatric malignancies with recurring translocations, copy number alterations, or mutations, including EWS (16–24).

Given the clinical need for a sensitive and specific biomarker in the monitoring of pediatric and adolescent patients with EWS, we developed a clinically feasible, ctDNA-based workflow. Here, we present the data of a first set of patients from a single center and demonstrate clinical utility for ctDNA quantification in the clinical routine of high-risk EWS.

## Patients and methods

### Patients

**FIGURE 1:**
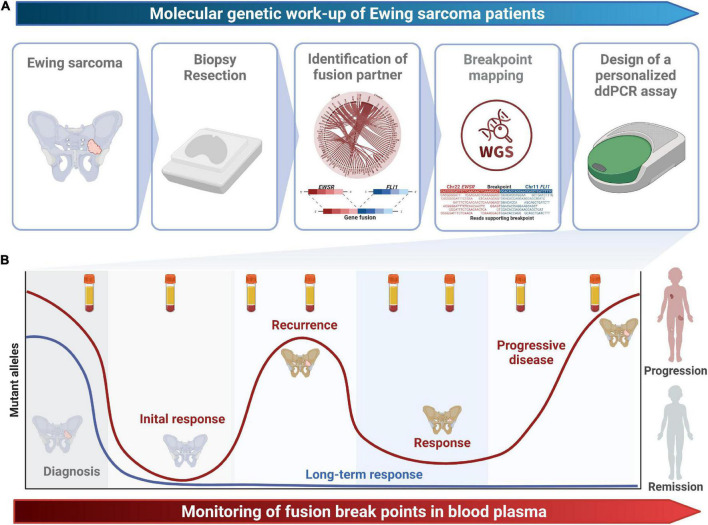
Study design. **(A)** The molecular genetic work-up from tissue sampling to the design of a patient-specific ddPCR assays spanning the fusion breakpoint is shown. **(B)** Longitudinal monitoring of fusion breakpoints in blood plasma enables early detection of recurrence and progression and might guide treatment decisions.

**FIGURE 2:**
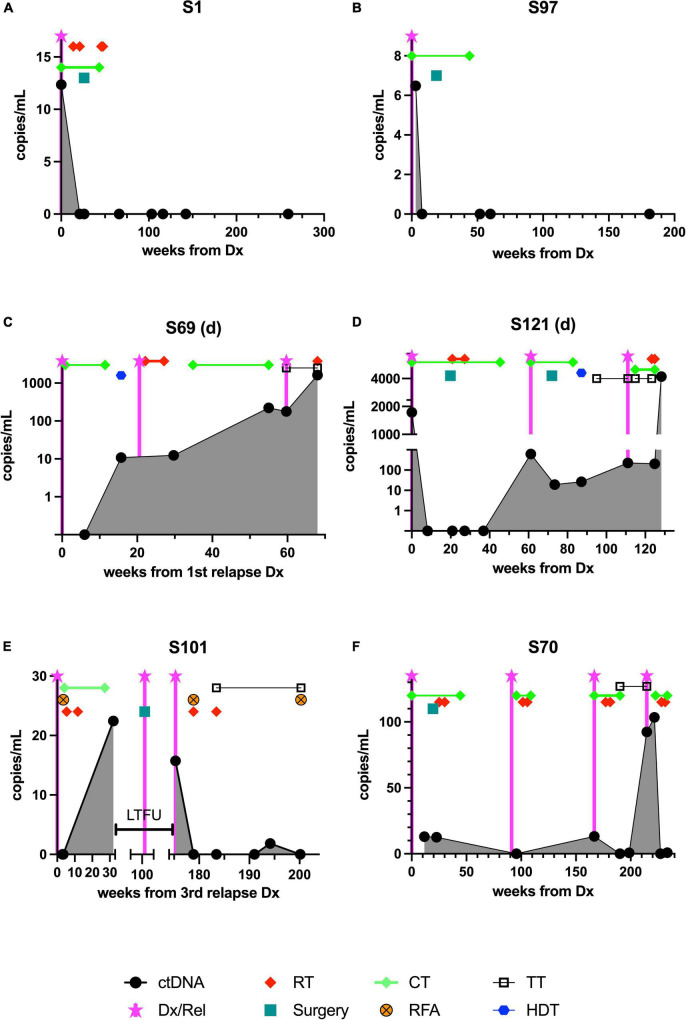
The course of ctDNA detected by ddPCR in 6 patients with Ewing sarcoma during treatment and follow-up. Samples were obtained and analyzed as outlined in section “Patients and methods.” Although the time points were not standardized, the aim was to obtain a baseline sample, one taken during neoadjuvant chemotherapy, one taken during adjuvant therapy, and multiple samples taken at time points during follow-up. **(A,B)** The upper panel shows the time course of two patients in first complete remission (CR1), **(C,D)** the center panel shows results from two patients who died from relapse, and **(E,F)** the lower panel shows results from two patients currently receiving relapse therapy. ctDNA, cell-free tumor DNA; Dx/Rel, time point of initial or relapse/progression diagnosis; RT, radiotherapy; CT, chemotherapy; RFA, radiofrequency ablation; TT, targeted therapy; HDT, high-dose chemotherapy followed by autologous stem cell rescue; LTFU, time period when patient was lost to follow-up.

Between January 2016 and October 2021, 25 consecutive patients were admitted with a diagnosis of a bone or soft tissue sarcoma to the Division for Pediatric Hemato-Oncology, Department of Pediatrics and Adolescent Medicine, Medical University of Graz. Of these, 17 (68%) had an EWS-translocation-positive tumor. To demonstrate the feasibility of our MRD detection and monitoring approach for routine clinical use, we preselected cases with different clinical courses/outcomes and a high likelihood of ctDNA detection. Based on the initial tumor burden, high-risk (i.e., metastatic or recurring) disease, or long-term duration of follow-up, six patients were selected for whole genome sequencing (WGS) of their tumor tissue to define the *EWS-FLI1* breakpoint. Blood samples were obtained from initial or relapse diagnosis, ideally before or during the first cycle of neoadjuvant chemotherapy, before surgery or radiotherapy, and at multiple time points during follow up (Figures 1, 2). However, in some patients, no pretreatment sample could be collected due to logistics issues (patient admitted from external institution, delay of informed consent, delay of sample collection, availability of specific tubes used in this study). Clinical data were derived from a retrospective chart review. Written informed consent was obtained from all patients or their legal representatives, and the study was performed under the current *Good Clinical Practice* and data protection guidelines with the approval of the institutional review board (IRB00002556; 28-397ex15/16).

### Figures

Figures were designed using *GraphPad Prism* version 9 (GraphPad Software, La Jolla, CA, United States) and *Biorender.com* (Toronto, ON, Canada; 2022).

### Molecular pathological analyses

#### Blood collection and cell-free DNA extraction

Blood was drawn into PAXgene Blood ccfDNA Tube (QIAGEN, Hilden, Germany) or Cell-Free DNA BCT tubes (STRECK, La Vista, NE, United States). Plasma was extracted as described previously (13) and stored at –80°C prior to analysis. Briefly, cfDNA was extracted from 1 or 2 ml of plasma using QIAamp Circulating Nucleic Acid Kit (QIAGEN) according to manufacturer’s protocol. DNA was quantified using Qubit dsDNA HS Assay Kit (Thermo Fisher Scientific, Waltham, MA, United States). cfDNA concentration ranged from 8.1 to 209.1 ng/ml plasma with an average of 38.3 ng/ml.

#### DNA/RNA extraction from tumor tissue

DNA and RNA were each extracted from 40 μm of unstained formalin-fixed and paraffin-embedded FFPE sections (16 unstained FFPE slides). The first and last 5 μm section was stained using hematoxylin and eosin and reviewed by a pathologist to assess tumor content in% according to a defined area and to ensure tumor content in deeper levels. If required (tumor content < 20%), a selective macro-dissection of a sample area enriched for the tumor was performed. Nucleic acid extraction was performed using the Maxwell RSC FFPE DNA Kit and the Maxwell RSC RNA FFPE kit (both Promega, Madison, WI, United States) according to the manufacturer’s recommendations. DNA was quantified using the Quantifluor ONE dsDNA system (Promega), while RNA concentration was determined by ribogreen fluorescence.

#### Archer fusion plex sarcoma panel

Archer fusion plex sarcoma panel (ARCHERDX, Invitae, Boulder, CO, United States) is a targeted RNA sequencing NGS panel capable of detecting known and novel fusions in target genes. For the current analysis, the following genes were used for fusion/rearrangement/translocation detection: *ALK, BCOR, BRAF, CAMTA1, CCNB3, CHMP2a, CIC, EPC1, EWSR1, FOSB, FOXO1, FUS, GLI1, HMGA2, JAZF1, MEAF6, MKL2, NCOA2, NTRK1, NTRK2, NTRK3, PAX3, PDGFB, PLAG1, RAB7a, ROS1, SS18 (SYT), STAT6, TAF15, TCF12, TFE3, TFG, USP6, VCP*, and *YWHAE*, applying unidirectional gene-specific PCR and NGS sequencing. In total, 250 ng of RNA were used for the Archer Fusion Plex Sarcoma Kit, and NGS libraries were sequenced on Ion Torrent Proton or Ion Torrent S5 using the applicable 200-bp sequencing chemistry (Thermo Fischer). Bioinformatic analysis was performed with ArcherDX Analysis software version 5.1.3. These analyses were performed in the routine diagnostic workflow of the Diagnostic and Research Institute of Pathology, Medical University of Graz (Graz, Austria).

#### Breakpoint mapping by whole genome sequencing

To pinpoint the exact genomic position of the breakpoints of the translocation identified with the AFPSP, WGS was performed. The quantity of genomic DNA was assessed with the Qubit 2.0 Fluorometric Quantitation system (Thermo Fisher Scientific), and NGS libraries were prepared from 50 ng of input material using the Illumina DNA Prep Kit (Illumina, San Diego, CA, United States). Sample-specific NGS libraries were pooled in an equimolar fashion, quality-checked on a 2100 Bioanalyzer automated electrophoresis instrument (Agilent, Santa Clara, CA, United States), and diluted before loading. A pool of 15 WGS libraries was sequenced on a NovaSeq S4 flow cell (Illumina, San Diego, CA, United States) with paired reads of 151 bp yielding 600–800 million reads per sample. Sequencing reads were aligned to the human reference genome (GRCh38).

#### Structural variant calling

Breakpoint mapping was performed using SvABA (25) with default parameters in the tumor-only mode. The genomic breakpoint of the known fusion gene was extracted from the SvABA result file and verified by visualizing the breakpoint reads in the integrative genomics viewer (IGV) (26).

#### Breakpoint junction PCR

Primers flanking the individual fusion breakpoints were designed using Primer3.^1^ Briefly, sequences upstream and downstream of the identified breakpoint were retrieved with the USCS browser^2^ and joined to generate a synthetic reference for primer design. The synthetic reference was loaded into Primer3, and the exact breakpoint was marked. Primers were selected up- and downstream of the breakpoint, while an internal fluorescent probe was placed overlapping the breakpoint with 2–6 3′ bases. Primers and probes were ordered from Eurofins GmbH (Austria) using an FAM fluorescence label and BH quencher on the probes. PCR assays were initially tested for specificity and sensitivity on tumor samples from the respective patients. The amplicon length was validated using agarose gels, and limiting dilution ddPCR was performed using a QX200 DropletDigital PCR instrument (BioRad). In addition, a second assay located on chromosome 2p14 was designed to count total genome equivalents. The ddPCR reaction was performed an initial denaturation of 96°C for 10 min, followed by 40 cycles of denaturation at 94°C for 30 s, combined annealing and extension at the temperature indicated in Table 1 and a post cycling step of 98°C for 10 min and final hold at 4°C. The numbers of droplets for each sample were over 12,000, and the ddPCR for each sample was performed in duplicates. For each run, a negative sample (from a healthy donor), a highly diluted positive control, and a no-template control sample were included. Raw fluorescence values were exported from the QX200 data files and processed using the ddpcr R package.^3^ Final results were reported as the allelic fraction of fusion positive genome equivalents and as the number of fusion positive molecules per milliliter of initial patient plasma.

**TABLE 1:** *EWS-FLI1* breakpoint definition and primers.

	EWSR breakpoint	FLI1 breakpoint	EWSR ddPCR primer	FLI1 ddPCR primer	Orientation	Fluorescent probe (3′-FAM + 5′-BHQ)	Annealing temp. [°C]
S1	[chr22:29288203]G	T [chr11:128806738]	AGTTCTTCTGTATGGAGAGAGGT	TGATGGTACTGAGGCTGTGG	fwd	ACCAGGAAGCAGCTGATCTT	58
S97	[chr22:29292655]T	G [chr11:128795030]	TGTGGGGTTGTTAAGGTCAGT	TTGAAACAGGGCCTCACTCT	fwd	TTTAAACCACAGAGTGCGCC	58
S69	G [chr22:29289258]	[chr11:128777239] A	CAGTAGGAAGTGAGCCCATAAT	TCGAAGAAACGGAGGGCG	rev	ACTATAGCCTGAGGTGCACC	58
S121	G [chr22:29288035]	[chr11128796041] A	GTGTTTTGGTTACCTCTCTCCA	CCCCTGCCAAGTATCTACCT	rev	CCTCCTCACAGAATATTTGCAGT	58
S101	C [chr22:29287795]	[chr11:128786094] G	TCTCAAGTGATCCTCCTGCC	CTGATGCCCAAGTGCCAAAA	rev	ATTCAGAACCTCGTGGGGAG	55
S70	C [chr22:29287723]	[chr11:128797267] T	TGACTGATAGGGAGGCCAAA	CGTGAATCCAAGACCACAGAC	rev	TGGGGAAGTTGTATGCAGTGA	55

## Results

### Breakpoint definition and workflow for cell-free circulating tumor DNA detection

The workflow for the individual fusion gene breakpoint definition and the subsequent quantitative detection of ctDNA in peripheral blood plasma is outlined in Figure 1. Initial RNA-based analyses allowed the identification of fusion gene exons involved in the translocation event. WGS was performed from tumor tissue to sequence the adjacent introns and to detect the exact genomic coordinates of the individual genetic breakpoint for each tumor. Primers for ddPCR assays were designed up- and downstream of the breakpoint and validated on tumor DNA (Table 1). By taking this approach, the initial setup of a patient-specific ctDNA assay could be performed within 3–5 weeks after obtaining tumor tissue (i.e., typically from the time point of the first biopsy). Once the assay was established, online real-time detection of ctDNA was feasible within 2–3 days.

### Clinical patient and tumor characteristics

The main clinical patient and tumor characteristics are presented in Table 2.

**TABLE 2:** Patient and tumor characteristics.

	S1	S97	S69	S121	S101	S70
Age at Dx (years)	9	7	10	18	19	3
Follow up duration (years after initial Dx)	5.5	4	5	2.5	13	4.5
Remission/living status	Alive, CR1	Alive, CR1	Deceased after early relapse, progressive disease	Deceased after early relapse, progressive disease	Alive, partial response after 5th relapse	Alive, partial response after 3rd relapse
Fusion gene	*EWS-FLI1*	*EWS-FLI1*	*EWS-FLI1*	*EWS-FLI1*	*EWS-FLI1*	*EWS-FLI1*
Initial/primary tumor volume (ml)	300	83	100	n.d.	n.d.	72
Initial/primary tumor location	Femur	3rd rib	Tibia + skip lesion	Femur + local metastases to pelvic bones and muscles	Multifocal bone (spine, pelvis, ribs, skull)	7th rib (ruptured hemato-thorax)
Initially detectable distant metastases	Lungs	None	None	None	Yes	None
Regression grade (Salzer Kuntschik)	II	I	I	II	V	V
Regression grade (vital cells)	Few	None	None	Few	> 50%	>50%
Primary tumor surgical margins	Free	Free	Free	Free	Free	Affected
Relapse			Multiple, bone	Multiple, bone	Multiple, bone	Multiple, bone
Chemotherapy (Euro-EWING or EWING2008)	X	X	X	X	X	X
Radiotherapy	X		X In relapse	X In relapse	X	X
Surgery	X	X	X	X	X	X
High-dose therapy and autologous stem cell rescue			X In relapse	X In relapse	X	
Additional alternative therapies	0	0	X In relapse	X In relapse	X	X In relapse
Number of ctDNA samples obtained	8	5	6	11	9	10

CR1, first clinical remission.

### In patients with durable response cell-free circulating tumor DNA declines and remains undetectable

As expected, in two patients with good response and continuous complete remission, ctDNA was detectable only at the time of diagnosis (Figures 2A,B).

#### Patient S1

This girl was diagnosed at the age of 9 years with a tumor of the right femur with an approximate volume of 300 ml and multiple bilateral lung metastases. At this time, 12.4 breakpoint-spanning copies/ml were identified in plasma. She was treated according to EWING2008 with polychemotherapy and radiotherapy (54 Gy right femur; 15 Gy lungs) and surgical resection (endoprosthetic implant; regression grade Salzer Kuntschik II). After 4.9 months, no ctDNA could be detected anymore and ctDNA remained undetectable throughout follow-up, which is in accordance with an ongoing oncological remission for 5.5 years (Figure 2A).

#### Patient S97

This boy suffered from Ewing sarcoma of his right 3rd rib with an initial volume of 83 ml without signs of metastases at the age of nearly 8 years. ctDNA could be detected with 6.5 copies/ml, and became undetectable after 1.1 months (Figure 2B) which was also reflected by an excellent response to chemotherapy according to EWING2008. After undergoing thoracal surgery to remove the residual mass together with the 3rd and adjacent ribs (regression grade Salzer Kuntschik I) followed by receiving postoperative chemotherapy as well as zoledronic acid until 15 months after his diagnosis, he is in ongoing oncological remission with 4 years of follow-up.

### A molecular cell-free circulating tumor DNA recurrence indicates therapy failure and tumor recurrence

In patients with unfavorable clinical courses, ctDNA dynamics were more volatile (Figures 2C–F).

#### Patient S69

At the age of 11 years, this boy was treated for EWS of his right tibia of approximately 100 ml volume as localized disease apart from a skip lesion according to EWING2008 and suffered a traumatic pathological fracture during induction chemotherapy, when the tumor had to be resected before completion of the scheduled neoadjuvant therapy (regression grade Salzer Kuntschik I). He suffered from a multifocal relapse with multiple bone metastases (skull, pelvis, 7th thoracic vertebra and rib), 3 years later and 2.5 years after the end of the initial therapy. Despite relapse treatment (consisting of alternating cycles of irinotecan and temozolomide or cyclophosphamide and topotecan, high-dose therapy with melphalan and treosulfan with autologous stem cell rescue, radiotherapy with 45 Gy applied to multiple skeletal lesions, and a “maintenance therapy” with trabectedin and irinotecan) the disease progressed, and ctDNA concentrations increased steadily, which corresponded to clinically overt progression. Highly increased positivity of a sample (221 copies/ml) in week 55 after his first relapse diagnosis indicated imminent progression (3rd relapse), resembling a molecular presence of progressive disease already 4–5 weeks before clinical diagnosis of the progress. Treatment failure to further experimental treatment attempts (including checkpoint inhibition followed by a combination of crizotinib and vorinostat), was further reflected by a continuous increase of ctDNA to 1,623 copies/ml at the time of death 18 months after relapse diagnosis (Figure 2C).

#### Patient S121

This young woman presented with EWS at the age of 18 years; the primary tumor was localized in the right proximal femur with local metastasis to the right ramus superior ossis pubis involving the acetabulum (a total volume was not defined). She was treated with EWING2008 polychemotherapy and surgery (regression grade Salzer Kuntschik II). Although at the time of diagnosis, 1,731 copies/ml ctDNA could be detected, ctDNA was undetectable throughout 4 follow-up samples. Four months after the end of postoperative chemotherapy, she suffered from a very early multifocal relapse with a large metastasis of the skull and pleural seedings, which was also reflected by a molecular recurrence (620 copies/ml). This relapse was treated with 7 cycles of temozolomide-irinotecan, surgery, and radiotherapy (thoracal photon therapy 19.8–45 Gy and cranial photon irradiation 30.6–45 Gy), high-dose chemotherapy with autologous stem cell rescue and four cycles of trabectedin-irinotecan. Although an initial clinical response and decreasing ctDNA levels (20 and 27 copies/ml) were noted, multifocal skeletal progress of the disease occurred only 11 months later. The disease was rapidly progressing despite treatment with topotecan-cyclophosphamide, cabozantinib, and palliative radiotherapy, and the young woman died 2, 5 years after her first diagnosis. The ctDNA concentration at the time of death was exceptionally high with 4,204 copies/ml (Figure 2D).

#### Patient S101

The first manifestation of EWS in this young man was noted when he was 19 years old; as he suffered from a multifocal disease spreading to multiple vertebrae and adjacent ribs, the skull, and the right os ilium, with multiple foci in the right lung and lymph nodes (a volume could not be calculated). He was treated with polychemotherapy (Euro-EWING), radiotherapy (44.8 Gy to pelvic bones, skull, vertebrae), surgery where feasible (regression grade V), high-dose chemotherapy with stem cell rescue, and remained in remission until 4 years after the first diagnosis. He then suffered from recurring, multifocal relapses in shorter intervals (2, 3, 2, and 1 years apart) at sites mostly adjacent to the previously irradiated or surgically removed sites. At the end of treatment for his 3rd relapse, we detected 22.4 ctDNA copies/ml. The patient decided to discontinue follow-up visits at our clinic only informed us of an intermittent surgery for localized (4th) relapse, when he returned to our care with the diagnosis of his 5th multifocal relapse. At that time point we also clearly detected ctDNA positivity with 15.7 copies/ml (Figure 2E). After initiation of local and systemic treatment (radiotherapy, radio frequency ablation, and an oral multi-kinase inhibitor) ctDNA became undetectable. We then noted one borderline result (1.8 copies/ml ctDNA), potentially corresponding to a marginal PET-positivity adjacent to the latest radiofrequency-treated pelvic lesion. After another intervention with radio frequency ablation, no ctDNA could be detected any more, approximately 6 months post-diagnosis of his 5th relapse, and > 12, 5 years after his first diagnosis.

#### Patient S70

The girl was diagnosed with a localized 72 ml tumor of the 7th rib and seeding into a hematothorax due to spontaneous tumor rupture at the age of 3.5 years. She was treated with polychemotherapy according to EWING2008, undergoing a surgical *en-bloc* resection of tumor, ribs, parts of the vertebrae (regression grade V according to Salzer and Kuntschik and positive surgical margins), and local radiotherapy (45 Gy). Eleven months after the end of initial treatment she suffered from a local, unifocal relapse. Therapy was initiated with irinotecan and temozolomide, and the girl additionally received radiotherapy (proton therapy 45.9–51 Gy). Fifteen months after the first relapse, new distant metastases were diagnosed in neck vertebrae as well as in the os ilium and the 5th rib. This second, multifocal, relapse was treated with topotecan, cyclophosphamide, and proton radiotherapy (45–54 Gy) followed by a maintenance therapy with cabozantinib and newly achieved ctDNA plasma negativity. Five months later, a third, multifocal relapse accompanied by a rise of ctDNA to 92–103.5 copies/ml occurred while still under cabozantinib, prompting a switch to treatment with pegylated liposomal doxorubicin and vincristine (ongoing) and another attempt of local tumor eradication with radiotherapy (20 Gy photon therapy to the left femur, skull base, 10th rib paravertebral, 7th rib and pleura dorsal axillar line). These measures were again followed by a molecular ctDNA response (Figure 2F). She is currently without detectable active disease around 7 months after diagnosis of her third relapse.

## Discussion

The present liquid biopsy study in Ewing sarcoma conducted at a single center focused on the clinical implementation of MRD detection in Ewing sarcoma patients using ddPCR to track fusion breakpoints in blood plasma. To this end, WGS of the primary tumor was performed to obtain detailed, patient-specific molecular information about individual breakpoints of the fusion gene. Fusion breakpoint calling is highly facilitated by prior knowledge about fusion partners from the Archer panel. However, since 90% of Ewing’s sarcomas contain a *t*(11;22) (q24;q12) translocation which fuses the EWS gene on chromosome 22 with the FLI1 gene on chromosome 11, prior knowledge about fusion partners is not necessarily required for Ewing sarcoma. Yet, our approach is easily transferrable to all types of translocation-associated sarcomas.

Digital droplet PCR (ddPCR) assays spanning the fusion breakpoints can be designed based on patient-specific translocations to enable a highly sensitive and specific detection of tumor-derived molecules in blood plasma. Once the patient-specific breakpoint assay was established, the turnaround time required to quantify ctDNA in follow-up samples was short: 2–3 days. Our tumor-informed approach yielded clinically relevant results in concordance with the clinical courses of six consecutive EWS patients. Molecular ctDNA response correlated well with treatment response and ongoing remission, while increasing levels and ctDNA recurrence was associated with treatment failure and progression. We observed that repeatedly or newly detectable levels of ctDNA are a strong predictor of disease recurrence, as has been seen in many types of cancer (27). We also noticed a reliable intra-individual (longitudinal) decrease in ctDNA levels in response to clinically effective anticancer therapy according to Euro-Ewing or EWING2008 protocols, as previously reported (22). Thus, the relative courses of ctDNA concentrations in peripheral blood plasma correlated well with the clinical status and imaging results per individual (intra-individually). However, absolute ctDNA concentrations were not comparable between patients (inter-individually) and did not directly correlate with their different tumor burdens. Inter-patient variance of the maximum number of fusion breakpoints detected ranged from 6 to 4,000 copies/ml with the highest ctDNA concentrations seen in progressive, refractory disease (patients S69 and S121). Yet, the patient number of our cohort was too small to correlate absolute ctDNA concentrations to clinical risk factors or long term-outcome.

Larger studies of localized Ewing sarcoma and osteosarcoma demonstrated that the abundance of ctDNA molecules present at the time of diagnosis correlated with outcome (19, 22), and higher ctDNA concentrations of EWS during or after treatment were associated with known clinical risk factors such as larger initial tumor volume, pelvic or osseous localization, metastatic disease, higher age at diagnosis, and poor histological response to chemotherapy (22). Klega et al. showed that translocation detection in EWSR1 fusion gene-associated tumors yielded the most reliable MRD results for ctDNA detection, and ctDNA detection in various solid tumors correlated well to the clinical courses in children (18). In patients with metastatic soft tissue sarcoma, cfDNA and ctDNA levels positively correlated with the disease burden (28), whereas this correlation was not constantly observed in other patients with various types of non-metastatic soft tissue sarcoma (29), indicating the urgent need to optimize and standardize the technological methodology separately (specifically) for liquid biopsy detection in the clinical setting of each tumor entity.

For translocation-positive tumors, the main and constant driver is the fusion gene, which makes it a perfect candidate for reliable treatment monitoring. As previously proposed from encouraging results in xenograft studies for MRD monitoring in EWS (30), ddPCR appears to be the most adequate and cost-effective method today. Breakpoint spanning ddPCR is highly specific as the un-translocated DNA cannot give rise to the specific PCR products. Moreover, the detection of the fusion breakpoints using ddPCR is not hampered by any technical artifacts due to deamination of cytosine or sequencing noise connected to NGS based assays. However, although being highly specific and sensitive, ddPCR does not enable the identification of clonal resistance mutations or clonal evolution of the tumor. To this end, molecular barcoded NGS approaches may provide a more comprehensive view of the genetic events associated with tumor evolution and progression (31). Since the workflow presented here relies on WGS of the primary tumor tissue for establishing a patient-specific ctDNA assay, all other types of genetic alteration including single nucleotide variants, insertions-deletions, structural aberrations, and copy number alterations can be inferred from the same data set. Such an integrated genomic analysis additionally enables the establishment of mutational signatures that can also represent biomarkers indicative of therapy sensitivity, prognosis, and therapy contraindications or reveal novel biologic features sarcomas (32).

Based on the presented data, we are just about to implement our workflow in routine patient care. The fact that the Archer assay is performed within the routine diagnostic workup of sarcoma and informs about the fusion partner greatly facilitates the fusion calling, which in many instances is complicated by false positives caused by mapping ambiguity in repetitive regions, transcriptional read-through (33). In this work, we focused only on the known fusion event which defines the tumor entity and is thus the most useful target for ctDNA based treatment monitoring. It is of note, though, that third-generation nanopore sequencing might represent an alternative, simple and low-cost workflow for DNA translocation detection (34). More recently, a method combining target-selected and strand-specific CRISPR-Cas9 activity for fusion gene driver enrichment and long read nanopore sequencing was developed to *de novo* identify fusion partner or breakpoint-location with high confidence (35). However, the short-read based WGS approach presented in this work has the advantage that it is applicable to routine pathology samples which are usually formalin-fixed and paraffin-embedded. DNA extracted from these samples is highly fragmented and thus not desirable for long-read sequencing.

ctDNA data will be used as complementary information in routine clinical monitoring of EWS patients. Although two large studies in (127 and 199) patients with recurrence of rhabdomyosarcoma did not show a significantly improved overall survival upon imaging-based early detection of relapse vs. clinical symptom-based detection (36, 37), prevention of delay before further molecular and clinical progression occurs, potential additional mutations are acquired, or more metastases are seeded, appear mandatory. As ctDNA recurrence can often be observed before clinical progression/relapse is evident, it might open up a window of opportunity to treat patients while tumor burden and molecular heterogeneity are at their lowest (38–41). However, studies whether intervention based on a ctDNA recurrence actually increases the survival are only under way, and the actual clinical utility still needs to be proven in randomized trials. Recent reviews have also emphasized this point, and it is exemplified by both published and ongoing trials (22, 42, 43). We believe that MRD detection by ctDNA quantification will revolutionize the current post-treatment follow-up schedules, most of which currently rely on clinical symptoms observed in expansions and imaging studies; these have only limited resolution due to their size and specificity. Because they may involve the use of contrast agents, radiation, and, in younger children, anesthesia, the frequency of such restaging studies is limited by considerations of toxicity and tolerability. Having another specific and sensitive tool for EWS MRD monitoring on hand might eventually spare these children and adolescents from having to undergo (many of) these procedures.

## Data availability statement

The data that support the findings of these studies are available from the corresponding author upon request. BAM-files extracted from WGS spanning the breakpoint regions (20 Mb) have been deposited at the European Genome-phenome Archive (EGA; http://www.ebi.ac.uk/ega/), which is hosted by the EBI, under the accession number EGAS00001006433.

## Ethics statement

The studies involving human participants were reviewed and approved by the Ethikkommission, Medical University of Graz. Written informed consent to participate in this study was provided by the participants’ legal guardian/next of kin.

## Author contributions

MS wrote the first draft of the manuscript and designed Table 2 and Figure 2. KK, TM, AT, and EH performed molecular pathological analyses. EH designed Figure 1. KK made Table 1. BL-A provided results of molecular pathological and histopathological analyses and diagnoses. JS, AL, MS, and MB collected patient data and blood samples. All authors reviewed the manuscript and approved the submitted version.

## Footnotes

1
https://primer3.org/

2
http://genome-euro.ucsc.edu/index.html

3
https://cran.r-project.org/package=ddpcr
